# Analysis of Indonesian Spice Essential Oil Compounds That Inhibit Locomotor Activity in Mice

**DOI:** 10.3390/ph4040590

**Published:** 2011-04-06

**Authors:** Muchtaridi, Adjeng Diantini, Anas Subarnas

**Affiliations:** Faculty of Pharmacy, Universitas Padjadjaran, Jl KM 21.5 Bandung-Sumedang, Jatinangor, Indonesia; E-Mail: anassubarnas@unpad.ac.id (A.S.)

**Keywords:** Indonesian spices, locomotor activity, SPE, GC-MS

## Abstract

Some fragrance components of spices used for cooking are known to have an effect on human behavior. The aim of this investigation was to examine the effect of the essential oils of basil (*Ocimum formacitratum* L.) leaves, lemongrass (*Cymbopogon citrates* L.) herbs, ki lemo (*Litsea cubeba* L.) bark, and laja gowah (*Alpinia malaccencis* Roxb.) rhizomes on locomotor activity in mice and identify the active component(s) that might be responsible for the activity. The effect of the essential oils was studied by a wheel cage method and the active compounds of the essential oils were identified by GC/MS analysis. The essential oils were administered by inhalation at doses of 0.1, 0.3, and 0.5 mL/cage. The results showed that the four essential oils had inhibitory effects on locomotor activity in mice. Inhalation of the essential oils of basil leaves, lemongrass herbs, ki lemo bark, and laja gowah rhizomes showed the highest inhibitory activity at doses of 0.5 (57.64%), 0.1 (55.72%), 0.5 (60.75%), and 0.1 mL/cage (47.09%), respectively. The major volatile compounds 1,8-cineole, α-terpineol, 4-terpineol, citronelol, citronelal, and methyl cinnamate were identified in blood plasma of mice after inhalation of the four oils. These compounds had a significant inhibitory effect on locomotion after inhalation. The volatile compounds of essential oils identified in the blood plasma may correlate with the locomotor-inhibiting properties of the oil when administered by inhalation.

## 1. Introduction

The fragrance of essential oils of aromatic plants is known to influence locomotor activity [1-4]. Kovar *et al.* [5] investigated the activity of essential oil of rosemary and its main constituent, 1.8-cineole in this context. Locomotor activity of the test animals increased significantly after they inhaled the oil. Furthermore, Buchbauer *et al.* [6] reported that 40 fragrances and six essential oils from Europe were observed to have the ability to reduce the locomotor activity of test animals. Studies conducted by Sangat and Roematyo [7] in the field of etnopharmacology indicated that some 49 species of Indonesian plants from 22 families are traditionally used in aromatherapy, but publications of data, especially those concerning active compounds of plant volatile oils responsible for the activity are very rare. We have previously studied essential oils of nutmeg seeds that showed a higher inhibitory effect on locomotor activity than lavender oils. Inhalation at a dose of 0.5 mL decreased locomotion by 68.62%, while the doses of 0.1 mL and 0.3 mL inhibited locomotion by 62.81% and 65.33%, respectively [8]. In this study, we have investigated the effect of essential oils of four Indonesian spices, basil (*Ocimum formacitratum*), lemongrass (*Cymbopogon citratus.*), ki lemo (*Litcea cubeba* L.) and laja gowah (*Alpinia malaccencis* Roxb.) on locomotor activity in mice.

In the West Java region of Indonesia, basil is used as a salad and seasoning in chicken steam processing and lemongrass, which has strong fragrance, is commonly used to reduce fishy smells in food processing. Ki lemo barks are used as a flavor enhancer in soup by the Sudanese people in West Java, whereas laja gowa, which is usually applied as spice in meat processing, is also widely used as a traditional antiemetic medicine. In addition the essential oils of the four spices are commonly used in aromatherapy as massage oils or by inhalation [9]. In Europe and the US [10,11], basil leaf oils are commercialized as aromatherapeutic materials and the citronella oil of lemongrass is also used in aromatherapy. Thesse properties led us to investigate those four essential oils for their pharmacological activity and active components.

As in our previous study the essential oil of nutmeg seeds caused inhibition of locomotor activity [8], this investigation has been conducted to examine the inhibitory effects of the essential oils of the four spices, basil, lemongrass, ki lemo, and laja gowah, on locomotor activity in mice and identify the active component(s) that might be responsible for that activity. The essential oils were administered by inhalation at doses of 0.1, 0.3, and 0.5 mL/cage and the effect on locomotor activity was observed using a rotatory wheel cage method. The plasma concentrations of essential oil compounds were measured by off-line SPE and GC-MS.

## 2. Results and Discussion

### 2.1. Composition of Essential Oils

Essential oil of basil leaves contained a high content of geranial (19.3%) and linalool (8.17%) and that of lemongrass had α-citral (32.70%), β-citral (28.99%), linalool (1.6%), citronellal (0.65%) and methyl cinnamate (0.23 %). In the essential oil of ki lemo bark, 1.8-cineole (26.59%) and citonellol (21.69%) were the major components, whereas in that of laja gowah rhizomes, methyl cinnamate (64.4%), α-pinene (14.95%), β-pinene (12.44%) and 1,8-cineole (9.89%) were identified as the compounds with the highest levels.

### 2.2. Locomotor Activity of Mice after Inhalation of Essential Oils

In this study, the effect of essential oils of four Indonesian spices on locomotor activity in mice was compared with that caused by lavender oil. The latter was used as a positive control because it is reported to reduce the locomotor activity of female and male laboratory animals [4]. The locomotor activity data are shown in Table 1. As may be seen, in general the four essential oils have a significant locomotor inhibitory activity compared to control. Inhalation of basil leaves and lemongrass essential oils decreased mice locomotor activity in a dose-dependent manner, contrary to the effect seen with the other two oils. The essential oil of basil leaves at doses of 0.1, 0.3, and 0.5 mL decreased locomotor activity by 50.48, 55.33 and 58.36%, respectively, and that of lemongrass at the same doses caused a locomotor activity decreases of 45.21, 50.99 and 66.66%, respectively. These dose-dependent effects might be due to higher concentrations of active components in the higher doses. On the other hand, the ki lemo bark oil showed strong but not dose-dependent inhibitory effects, in that the dose of 0.3 mL had a lower effect (54.97%) than that of 0.1 (58.16%) and 0.5 mL (61.41%). Strange results were observed with the effect of the laja gowah rhizome oil, whereby increasing the doses decreased the inhibitory effect. A 0.1 mL dose gave the highest effect on locomotor activity and it might be the most effective dose for the tested oil.

### 2.3. Application SPE-GC/MS for Analysis of Lead Compounds in Blood Plasma of Mice after Inhalation of Essential Oils of Spices

In our previous study [8], we successfully applied SPE methods to isolate volatile compounds from blood plasma before detection and quantification of the compounds by GC-MS.

**Figure 1:**
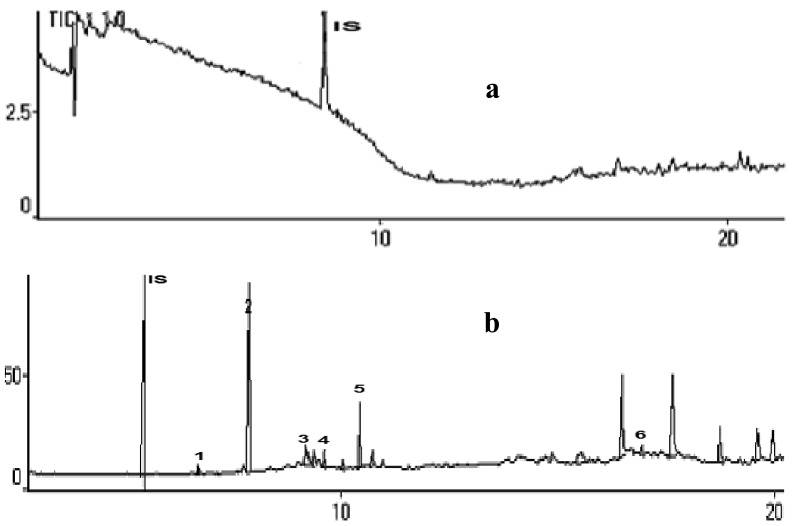
Total ion chromatogram of a blood plasma sample after 1 h inhalation of basil leaf oils. (**a**) sample preparation without SPE aid, no volatile compounds could be detected in blood plasma; (**b**) Sample preparation using SPE C-18, some volatile compounds were found in blood plasma. IS: Internal standard (1,4-dichlorobenzene): 1: 1,8-cineole; 2: linalool; 3: 4-terpineol; 4: α-terpineol; 5 : linalyl acetate; 6: α-humulene.

A mixture of methanol and water (60:40) was used for sample preparation to reduce solvent volume and time. The recovery of this analysis was increased to 90%. This method also increased reproducibility and recovery from the matrix and reduced interferences from the blood plasma matrix, as shown in Figure 1.

**Figure 2:**
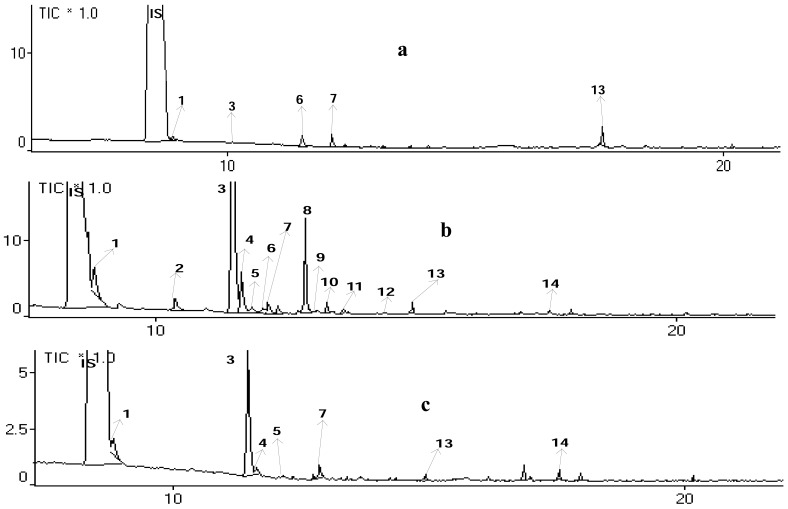
Total ion chromatogram of blood plasma sample after inhalation of ki lemo bark oils. (**a**). chromatogram of the sample after ½ h inhalation; (**b**). chromatogram of the sample after 1 h inhalation; (**c**). chromatogram of the sample after 2 h inhalation. IS: Internal standard (1,4-dichlorobenzene). Identity of peaks is shown in Table 5.

In addition, reproducibility was shown consistently on replication of the analysis in which the retention times of each interesting compound were very similar. Figure 2 shows that three chromatograms generated by different treatments of blood samples gave the same ion chromatogram pattern after inhalation of ki lemo bark oils, and many volatile compounds could be detected. Compounds identified in blood plasma with high bioavailability after 30 to 120 min of exposure might be active compounds responsible for the observed locomotor effects and thus they might be interesting lead compounds [6].

#### 2.3.1. Basil leaf essential oil compounds detected in mice blood plasma after different inhalation durations

As shown in Table 2, compounds were identified in blood plasma after one and 2 h inhalation of essential oils of basil leaves. The main compounds identified were linalool, linalyl acetate, 4-terpineole and α-terpineol. They have been proven to show anxiolytic, anticonvulsant and sedative activity [6,12-15].

#### 2.3.2. Lemongrass herb essential oil compounds detected in mice blood plasma after different inhalation durations

Citronellal was a dominant compound in the blood plasma of mice after inhalation of essential oils of lemongrass herbs, as seen in Table 3, although in this oil the content of citronellal and methyl cinnamate is small.

This fact might be connected with the previous findings that the oils are absorbed into the blood through the lungs or skin, and citronellal at low concentrations (10 and 30 pM) potentiates the response in the presence of GABA, because they probably bind to the site of action in GABAA receptors and they have high affinity for the GABAA receptor [13].

#### 2.3.3. Laja gowa rhizome essential oil compounds detected in mice blood plasma after different inhalation durations

As shown in Table 4, the compounds 1,8-cineole and methyl cinnamate were found at very high concentrations in blood plasma after one and 2 h inhalation. However, α and β-pinenes, known as the major components in the essential oils of laja gowa rhizomes, were strangely not detected.

#### 2.3.4. Ki lemo bark essential oil compounds detected in mice blood plasma after different inhalation durations

**Figure 3:**
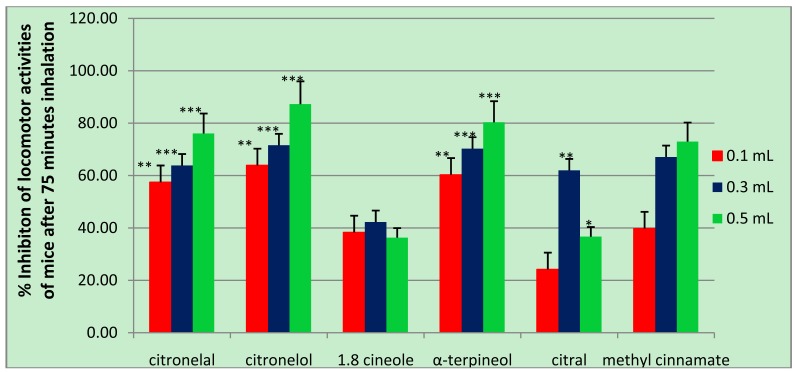
Bar graphs of the average number of locomotor activities of mice after 75 min inhalation of single detected compounds. * F_6,35_ = 3.30 P < 0.05, as compared with the contol treatment. (by ANOVA followed by Duncan *post hoc* test). ** F_6,35_ = 14.10 P < 0.01, as compared with the contol treatment. (by ANOVA followed by Duncan *post hoc* test). *** F_6,35_ = 26.10 P < 0.001, as compared with the contol treatment. (by ANOVA followed by Duncan *post hoc* test).

Citronelol, citronellal, α-terpineol, and 1.8-cineole were identified in blood plasma of mice after inhalation of ki lemo bark essential oil (Figure 3 and Table 5).

These results were in accordance with the previous study [13]. The mixture of 4-terpineol and cineole or citral give greater potentiation than that of citral, cineole, or butanol, but less than that caused by 4-terpineol [13,17]. Figure 2 shows that peaks no. 1 (1,8-cineole), 3 (citronellal), 7 (α-terpineol), and 8 (citronelol) appeared in the ½, 1 and 2 h inhalations. The level of those compounds in blood plasma was higher after 1-hour inhalation as compared with that after ½ and 2 h inhalation.

### 2.4. Locomotor Activity Effects of Single Identified Compounds in Mice

The dominant compounds, 1,8-cineole, α-terpineol, citronelol, citronelal, and methyl cinnamate were thought to have a role in inhibiting locomotor activity, thus, they we sought to examine them individually for their activity in mice. These compounds were all detected in more than one essential oil and at a concentration of more than 1 μg/mL. In this study, not all compounds identified in the four spices were tested for their activity, and 4-terpineol, for example, was not examined because it was already known to have up to 79% inhibitory activity on locomotion in mice [8]. For the examination of methyl cinnamate, 70% ethanol was used as control since methyl cinnamate was dissolved in 70% ethanol. As shown in Table 6 and Figure 3, all compounds tested significantly inhibited locomotor activity in mice in a dose-dependent manner, except 1.8 cineole and citral. Among them, citronelol gave the highest effect followed by α-terpineol, citronelal, and methyl cinnamate. Other two compounds, 1.8 cineole and citral, showed optimal inhibition at a dose of 0.3 mL, but for 1.8 cineole, the effects of its three doses were not significantly different according to ANOVA and Duncan *post hoc* test analysis.

These results suggest that all compounds might be responsible for the inhibitory activity on locomotor caused by the four spices tested. In the basil sample, α-terpineol and 1.8 cineole were thought to cause inhibition in locomotor activity in addition to 4-terpineol, as reported [8]. In the other three spices, the compounds responsible for the inhibitory activity might be citronelal, methyl cinnamate, and citral for lemongrass, citronelol, citronelal, and 1.8 cineole for ki lemo, and methyl cinnamate and 1.8 cineole for laja gowa. This assumption was based on the fact that those active compounds were contained in the corresponding spice plant. However, other compounds present in the four spice plants probably have the inhibitory activity because as mentioned above, that not all compounds contained in the four spice plants were tested for the activity. This study reported that the volatile compounds detected in blood samples might be related to the observed depressed locomotor activity in mice. This hypothesis was supported by the facts that depressed locomotor activity caused by essential oil is due, at least in part, to a direct pharmacological action of one or more of its constituents [8,13].

## 3. Experimental

### 3.1. Materials

#### 

*Spice materials*: The plant materials used were basil leaves (*Ocimum formacitratum* L.) obtained from the Cileunyi traditional market, lemongrass herbs (*Cymbopogon citratus* L.) and laja gowah (*Alpinia malaccencis* Roxb.) rhizome from Tanjungkerta, Sumedang, and ki lemo (*Litcea cubeba* L.) bark from Lembang, West Java. Specimens were identified by the Herbarium Laboratory, Department of Biology, Faculty of Mathematical and Natural Sciences of Universitas Padjadjaran and voucher specimens are deposited at the Herbarium of the Department of Biology of the Faculty of Mathematic and Natural Sciences, Universitas Padjadjaran.

#### 

*Animals*: Male mice weighing 25 to 30 g and 2 to 3 months old were used. The mice were adapted for one week to the laboratory in which locomotor activity experiments were conducted and were selected for wheel rotations of between 150 to 300 rpm before the experiments were started.

#### 

*Chemicals*—Methanol puriss. p.a. (Merck) was used as eluent for SPE. Heparin tubes (Boehringer) were used for blood collection. Pure lavender (*Lavandula officinalis*) oils were obtained from Martina Bertho. C_8_-C_20_, C_21_-C_40_ alkane standards, 1,4-dichlorobenzene and methyl cinnamate were obtained from Sigma. Citronelal, citronelol, 1,8-cineole, and α-terpineole were purchased from Dragoco.

### 3.2. Methods

#### 3.2.1. Isolation of essential oils

Dried samples of each plant (500 g) were submitted to water-distillation in Monaco Lembang, West Java, for 3 h to isolate the essential oils. The oils were stored at −20 °C after the addition of sodium sulphate. Essential oils of basil leaves, herbal lemongrass, laja gowah rhizome, and ki lemo bark were obtained at 0.07%, 0.94%, 1.22%, and 1% yield, respectively.

#### 3.2.2. Mouse locomotor activity tests

Locomotor activity of mice was measured using a wheel cage, in which mice ran and the number of rotations was recorded by a meter. Cage inhalators contained a glass fiber (20 cm × 20 cm × 30 cm) and were equipped with an electric fan for the evaporation and distribution of volatile compounds. The mice were selected by weight (25 to 30 g) and by their ability to rotate the wheel cage up to 300 times in 30 min; eligible mice were then divided into three groups: a control group, a lavender oil group as positive control group (using 0.1, 0.3, and 0.5 mL/cage), and a essential oils tested as treatment group (using 0.1, 0.3, and 0.5 mL/cage). The application of the doses were based on the preliminary examination in which those doses were reasonable to be used and based on Kovar *et al.* [5]. Each group consisting of five mice was tested three separate times. After 30 min of inhalation, the mice were placed into the wheel cage and after 5 min; the number of rotations was recorded for 75 min in 15-minute intervals.

#### 3.2.3. GC/MS analysis

Analyses have been done according to a previously published method [8]. Measurements were performed using a Shimadzu QP-5050A gas chromatograph coupled to a VG Autospec Mass Spectrometer at 70 eV, 40–550 amu with a fused silica capillary column (DB-5MS, 30 m × 0.25 mm) using helium as a carrier gas and with temperature programming from 60 °C/5 min to 300 °C/min (10 °C/min) for blood plasma and 60 °C/5 min to 300 °C/2 min (10 °C/min) for essential oils. The MS was operated using an interface temperature of 240 °C, and an electron impact ionization of 70 eV with a scan mass range of 40–350 *m/z* (sampling rate of 1.0 scan/s).

#### 3.2.4. Qualitative analysis

Identification of the compounds was conducted by comparing their linear retention indices (LRI) with literature values and their mass spectral data with those from the MS data system (Wiley-229 lib, Nist-62 library and Nist-12 library) [16]. Linear retention indices were calculated using GC data of a homologous series of saturated aliphatic hydrocarbons (C_8_ to C_40_) separated on the same column using the same conditions as for GC analysis of the essential oils and the blood samples. The blood samples were collected from the corner parts of the eyes using capillary tubes and placed in a heparin tube. Blood samples were collected immediately after the mice inhaled the essential oil for a period of ½, 1 or 2 h.

#### 3.2.5. Quantitative analysis

Detailed analysis was performed using a modification of the methods in [5] and [18,19]. The blood samples (500 to 600 μL), obtained according to Jirovetz [18], were centrifuged (1,800 rpm/10 min) at room temperature and concentrated on a C_18_-column (100 mg). Volatile compounds were separated using a mobile phase of the mixture of methanol—bidistilled water (60:40). Five microlitres re-injected into the GC-MS. Quantification of the volatile compounds in the blood samples was accomplished using 1,4-dichlorobenzene 0.5% (500 μL) as an internal standard as according to the following [8] equation:

(1) $$\left[C\right]=\frac{A}{\text{IS}}\times \frac{\text{IS weight}\;(g)}{100\;\text{mL}}\times \%\text{EO}\times \text{IS volume}\times 10^{6}$$

where: *C* = concentration (g/g); *IS* = GC peak area of Internal Standard; *A* = GC peak area of compounds of essential oils; % *EO* = yield of essential oils.

#### 3.2.6. Statistical analyses

All locomotor activity test data are presented as mean ± S.E.M. Data were analyzed by ANOVA followed by Duncan *post hoc* test. Results were considered significant at *p* < 0.05. Data were analyzed using MINITAB^®^ 13.5 software.

## Figures and Tables

**Table 1:** Average number of mice wheel cage rotations within 75 min of inhalation of essential oils.

		**Average Numbers of Locomotors ± SD^a^**						**% Inhibitory effect**
		**Minutes**						
		**0–15**	**15–30**	**30–45**	**45–60**	**60–75**	**Average**	
**Controls**	**0**	280.4 ± 20.5	294.4 ± 4.3	311.4 ± 17.2	303.4 ± 14.6	297.4 ± 9.7	1,487	0
**Lavender oils**	**0.1**	217.6 ± 18.1	200.4 ± 12.3	195.6 ± 9.6	197.4 ± 7.9	193.2 ± 11.9	1,003.8	31.14 *
	**0.3**	187.8 ± 19.0	181.4 ± 20.5	186.2 ± 15.0	180.4 ± 16.4	178.4 ± 19.4	914.2	38.52 *
	**0.5**	139.6 ± 11.2	128.8 ± 10.9	116.8 ± 10.2	123.2 ± 8.9	118.2 ± 8.7	626.6	57.86 ***
**Basil leaf oils**	**0.1**	171.8 ± 53.3	151.2 ± 39.6	150.6 ± 52.2	130.4 ± 11.2	145.0 ± 42.8	749	49.63 *
	**0.3**	148.2 ± 24.2	134.2 ± 12.8	130.4 ± 26.3	136.2 ± 17.1	126.6 ± 13.1	675.6	54.57 **
	**0.5**	126.0 ± 11.9	145.2 ± 39.7	121.0 ± 25.0	116.6 ± 21.8	121.0 ± 20.4	629.8	57.64 ***
**Ki lemo bark oils**	**0.1**	129.0 ± 5.7	134.4 ± 9.7	132 ± 23.7	122 ± 19.7	115.4 ± 8.7	632.8	57.44 ***
	**0.3**	137.0 ± 20.2	143.4 ± 23.0	125 ± 12.5	139 ± 11.5	136.6 ± 19.8	681	54.20 **
	**0.5**	115.0 ± 9.2	123.8 ± 8.4	112.8 ± 10.7	113.8 ± 8.8	118.2 ± 11.2	583.6	60.75 ***
**Lemongrass herb oils**	**0.1**	166.1 ± 20.3	187.3 ± 22.5	161.5 ± 18.9	177.6 ± 22.4	136.1 ± 27.4	828.6	55.72 **
	**0.3**	148.5 ± 21.2	160.5 ± 25.5	145.3 ± 19.7	169.6 + 26.6	117.3 ± 30.2	741.2	49.85 **
	**0.5**	131.0 ± 19.6	121.8 ± 28.9	105.0 ± 17.8	54.17 ± 25.5	92.3 ± 28.6	504.27	33.91 *
**Laja gowah rhizome oils**	**0.1**	180.4 ± 24.8	174.3 ± 33.2	157.2 ± 29.7	146.8 ± 34.7	128.0 ± 22.9	786.7	47.09 **
	**0.3**	203.6 ± 40.1	196.5 ± 30.5	188.8 ± 19.7	171.9 + *3*6.6	147.2 ± 38.5	908	38.94 *
	**0.5**	246.6 ± 28.9	259.6 ± 38.7	198.7 ± 34.6	173.5 ± 30.9	183.4 ± 40.1	1,061.8	28.65 *

* F_6,35_ = 3.15 P < 0.05, as compared with the contol treatment. (by ANOVA followed by Duncan *post hoc* test);

** F_6,35_ = 6.05 P < 0.01, as compared with the contol treatment. (by ANOVA followed by Duncan *post hoc* test);

*** F_6,35_ = 14.26 P < 0.001, as compared with the contol treatment. (by ANOVA followed by Duncan *post hoc* test).

**Table 2:** Active volatile compounds identified in blood after inhalation of basil leaf essential oil.

**No.**	**Name**	**Inhalation**						***LRI* References ^a^**
		**½ h (R^c^ = 80 %)**		**1 hr (R ^c^ = 88 %)**		**2 hr (R ^c^ = 88 %)**		
		**LRI Exp ^b^**	**Conc. μg/mL**	**LRI Eksp ^b^**	**Conc. μg/mL**	**LRI Eksp ^b^**	**Conc. μg/mL**	
1.	1,8-Cineole	*nd*	*nd*	1,035	0.8	*nd*	*nd*	1,033
2.	Linalool	*nd*	*nd*	1,090	25.8	1,090	5.9	1,098
3.	Borneol	*nd*	*nd*	1,159	2.1	*nd*	*nd*	1,156
4.	4-Terpineol	*nd*	*nd*	1,166	3.9	1,165	1.9	1,177
5.	α-Terpimeol	*nd*	*nd*	1,178	2.6	1,178	1.3	1,189
6.	Linalyl acetate	*nd*	*nd*	1,216	9.6	1,216	0.6	1,257
7.	α-Humulene	*nd*	*nd*	*nd*	*nd*	1,446	2.2	1,454

*nd* = not detected;

a LRI reference in Adams [16] with a DB5 column;

b LRI experiment with DB5-MS column;

c Recovery (n = 2) was calculated on the basis of comparison between 1,4-dichlorobenzene (methanol diluted) in blood plasma and 1,4-dichlorobenzene in methanol only.

**Table 3:** Active volatile compounds identified in blood after inhalation of lemongrass steam essential oils.

**No.**	**Name**	**LRI Exp ^b^**	**Inhalation concentration (μg/mL)**			**LRI References ^a^**
			**½ h (R^c^ = 82%)**	**1 hr (R^c^ = 87%)**	**2 hr (R^c^ = 90%)**	
1.	Linalool	1,098	*nd*	*8.7*	*3.4*	1,098
2.	Citronellal	1,153	*nd*	122.5	8.9	1,156
3.	Citral	1,238	*nd*	4.7	*4.6*	1,240
4.	Methyl cinnamate	1,388	*nd*	11.5	*3.2*	1,379

*nd* = not detected;

a LRI reference in Adams [16] with DB5 column;

b LRI experiment with DB5-MS column;

c Recovery (n = 2) was calculated on the basis of comparison between 1,4-dichlorobenzene (methanol diluted) in blood plasma and 1,4-dichlorobenzene in methanol only.

**Table 4:** Active volatile compounds identified in blood after inhalation of laja gowa rhizome essential oils.

**No.**	**Name**	**LRI Exp ^b^**	**Concentration (μg/mL)**			**LRI References ^a^**
			**½ h (R ^c^ = 83%)**	**1 hr (R ^c^ = 85%)**	**2 hr (R ^c^ = 83%)**	
1.	1,8-Cineole	1,033	*nd*	122.5	8.9	1.156
2.	Methyl cinnamate	1,376	*nd*	140.5	98.9	1.379
3.	Methyl hexadecanoate	1,779	*nd*	nd	0.09	1.961
4.	Methyl octadecanoate	2,049	*nd*	0.69	0.90	2.200

*nd* = no detected;

a LRI reference in Adams [16] with DB5 column;

b LRI experiment with DB5-MS column;

c Recovery (n = 2) was calculated on the basis of comparison between 1,4-dichlorobenzene (methanol diluted) in blood plasma and 1,4-dichlorobenzene in methanol only.

**Table 5:** Active volatile compounds identified in blood after inhalation of ki lemo bark essential oil.

**No.**	**Name**	**LRI Exp ^b^**	**Concentration (μg/mL)**			**LRI References ^a^**
			**½ h (R ^c^ = 82%)**	**References^a^**	***2 hr*(R ^c^ = 83%)**	
1.	1,8-Cineole	1,032	5.5	59.9	14.3	1,033
2.	Linalool	1,098	*nd*	10.4	*nd*	1,098
3.	Citronellal	1,153	14.9	39.3	37.1	1,156
4.	*neo*-Isopulegol	1,161	*nd*	35.6	*nd*	1,145
5.	Isopulegol	1,171	*nd*	6.9	0.5	1,146
6.	4-Terpineol	1,180	*nd*	4.2	*nd*	1,181
7.	α-Terpineol	1,196	8.1	5.6	*nd*	1,189
8.	Citronellol	1,225	22.3	53.1	33.8	1,228
9.	Neral	1,238	*nd*	4.7	*nd*	1,240
10.	Linalyl acetate	1,246	*nd*	1.5	*nd*	1,257
11.	Nerol	1,249	*nd*	6.5	*nd*	1,228
12.	Geranial	1,267	*nd*	2.9	*nd*	1,270
13.	β-Terpenyl acetate	1,347	*nd*	5.7	0.9	1,350
14.	(E)-Caryophylene	1,427	*nd*	1.0	0.6	1,418

*nd* = not detected,

a LRI reference in Adams [16] with DB5 column,

b LRI experiment with DB5-MS column,

c Recovery (n = 2) was calculated on the basis of comparison between 1,4-dichlorobenzene (methanol diluted) in blood plasma and 1,4-dichlorobenzene in methanol only.

**Table 6:** The Average number of mice wheel cage rotations within 75 min of inhalation of f volatile compounds detected.

**Treatment**	**Doses (mL)**	**Mean Instead of Number of Average (minutes)**							**Inhibitory Effect (%)**
		**0–15**	**15–30**	**30–45**	**45–60**	**60–75**	**75–90**	**Total Number**	
**Normal control**	**Control**	373.2 ± 41.2	387.5 ± 32.3	253.5 ± 22.5	302.4 ± 38.5	213.2 ± 28.6	255.46 ± 19.34	1785.26	0
	**0.1 mL**	134.5 ± 12.1	128.9 ± 17.6	123.4 ± 19.6	130.5 ± 11.2	123.6 ± 12.4	118.4 ± 15.4	759.30	57.47 **
**Citronelal**	**0.3 mL**	120.3 ± 9.5	120.4 ± 15.4	118.3 ± 12.7	107.4 ± 16.4	88.9 ± 13.2	90.2 ± 13.0	645.50	63.84 ***
	**0.5 mL**	110.2 ± 8.9	118.9 ± 9.1	67.1 ± 15.6	70.2 ± 7.5	40.6 ± 7.2	20.2 ± 3.2	427.20	76.07 ***
	**0.1 mL**	119.2 ±29.8	115.4 ± 35.7	118.7 ± 25.7	102.3 ± 20.1	89.7 ± 18.6	99.3 ± 10.8	644.60	63.89 **
**Citronelol**	**0.3 mL**	100.3 ±10.5	98.9 ± 26.3	83.2 ± 14.4	90.2 ± 18.5	78.8 ± 16.1	56.3 ± 15.8	507.70	71.56 ***
	**0.5 mL**	57.2 ±8.8	48.6 ± 7.6	50.3 ± 7.5	32.2 ± 7.5	18.9 ± 4.8	20.2 ± 1.3	227.40	87.26 ***
	**0.1 mL**	223.2 ±27.6	189.2 ± 24.3	190.3 ± 29.8	180.7 ± 35.1	157.5 ± 26.4	160.6 ± 22.3	1101.50	38.30 *
**1.8-Cineole**	**0.3 mL**	200.2 ± 31.18	180.5 ± 27.5	167.8 ± 26.22	207.5 ± 12.2	144.3 ± 11.8	130.2 ± 16.54	1030.50	42.28 *
	**0.5 mL**	192.7 ± 34.8	187.8 ± 35.7	188.4 ± 36.8	179.4 ± 32.1	190.1 ± 31.3	198.7 ± 34.4	1137.10	36.31 *
	**0.1 mL**	120.3 ± 33.55	128.7 ± 23.4	119.8 ± 22.8	112.8 ± 32.3	117.7 ± 22.1	109.5 ± 32.32	708.80	60.30 **
**α-Terpineol**	**0.3 mL**	111.1 ± 11.2	98.8 ± 17.2	88.6 ± 12.7	99.2 ± 12.8	76.8 ± 18.1	56.2 ± 17.4	530.70	70.27 ***
	**0.5 mL**	99.8 ± 9.7	78.6 ± 12.5	60.4 ± 18.4	43.4 ± 12.7	38.7 ± 8.6	30.1 ± 6.8	351.00	80.34 ***
	**0.1 mL**	289.4 ± 38.5	300.8 ± 32.4	240.3 ± 42.1	196.1 ± 36.3	176.4 ± 29.8	150.4 ± 31.3	1353.40	24.19
**Citral**	**0.3 mL**	208.6 ± 31.18	108.2 ± 27.5	100.81 ± 26.22	96.6 ± 12.2	90.2 ± 11.8	74.1 ± 16.54	678.51	61.99 **
	**0.5 mL**	222.3 ± 31.18	220.4 ± 27.5	199.3 ± 26.22	200.3 ± 12.2	187.5 ± 11.8	100.6 ± 16.54	1130.40	36.68*
**70 %ethanol as control**	**0.1 mL**	292.2 ± 38.4	280.9 ± 37.2	300.2 ± 35.4	302.9 ± 36.5	280.4 ± 32.8	285.4 ± 34.1	1742.00	0
	**0.3 mL**	300.2 ± 38.1	304.4 ± 39.5	289.2 ± 35.5	290.8 ± 35.4	288.7 ± 30.8	302.3 ± 29.9	1775.60	0
	**0.5 mL**	311.2 ± 32.1	296.5 ± 38.7	296.3 ± 30.20	311.2 ± 32.5	300.2 ± 33.1	301.4 ± 35.6	1816.80	0
**Methyl cinnamate**	**0.1 mL**	250.2 ± 38.22	200.1 ± 33.4	180.7 ± 32.3	165.4 ± 25.5	120.5 ± 32.2	132.2 ± 37.5	1049.10	39.78 *
	**0.3 mL**	120.4 ± 28.8	100.2 ± 29.2	111.1 ± 25.7	98.8 ± 14.4	86.7 ± 10.6	67.4 ± 9.4	584.60	67.08 **
	**0.5 mL**	98.6 ± 31.18	99.8 ± 27.5	90.7 ± 26.22	90.1 ± 12.2	67.7 ± 11.8	44.4 ± 16.54	491.30	72.96 ***

* F_6,35_ = 3.30 P < 0.05, as compared with the contol treatment. (by ANOVA followed by Duncan *post hoc* test).

** F_6,35_ = 14.10 P < 0.01, as compared with the contol treatment. (by ANOVA followed by Duncan *post hoc* test).

*** F_6,35_= 26.10 P < 0.001, as compared with the contol treatment. (by ANOVA followed by Duncan *post hoc* test).
